# Correction to “Praziquantel Nanoparticle Formulation for the Treatment of Schistosomiasis”

**DOI:** 10.1021/acsanm.5c02476

**Published:** 2025-06-06

**Authors:** Ana C. Mengarda, Bruno Iles, Vinícius C. Rodrigues, Ana L. L. do Nascimento, Victoria P. Machado, Walberson S. Reatgui, Patrícia S. Bento, Marina A. Radichi, Taís C. Silva, Fernanda S. Teixeira, Maria C. Salvadori, Marina M. Simões, Karen L. R. Paiva, Cesar K. Grisolia, Maria L. Fascinelli, Sebastião W. Silva, Marcílio S. S. Cunha-Filho, Sônia N. Bao, João P. F. Longo, Josué de Moraes

In our original article, the Supporting Information PDF file is not correct. The correct Supporting Information PDF file is associated with this Addition and Correction.

## Supplementary Material



## Notes

Composition of the mixtures, stability of potential zeta, DSC results for PZQ, stability of SNEDDS BLANCK, release kinetic equations, and rheology results (PDF)

