# Supplementary material for: Correction to “Praziquantel Nanoparticle Formulation for the Treatment of Schistosomiasis”
Source: ACS Appl Nano Mater. 2025 Jun 6;8(22):11730. doi: 10.1021/acsanm.5c02476 (PMC12150259; doi:10.1021/acsanm.5c02476)
Supplement: Supplementary file 1 [file an5c02476_si_001.pdf]

Supporting Information

Supplementary Material – Correction

Correction to 'Praziquantel Nanoparticle Formulation for the Treatment of Schistosomiasis'

Ana C. Mengarda[1,2,†], Bruno Iles[3,†], Vinícius C. Rodrigues[1], Ana L. L. do Nascimento[4], Victoria P. Machado[3], Walberson S. Reatgui[3], Patrícia S. Bento[3], Marina A. Radichi[5], Taís C. Silva[1], Fernanda S. Teixeira[6], Maria C. Salvadori[6], Marina M. Simões[7], Karen L. R. Paiva[7], Cesar K. Grisolia[3], Maria L. Fascinelli[3], Sebastião W. Silva[7], Marcílio S. S. Cunha-Filho[4], Sônia N. Bao[5], João P. F. Longo[3,*], Josué de Moraes[1,2,8*]

[1] Núcleo de Pesquisa em Doenças Negligenciadas, Universidade Guarulhos, Guarulhos, SP, Brazil.

[2] Instituto de Ciências Biomédicas, Universidade de São Paulo, São Paulo, SP, Brazil.

[3] Departamento de Genética e Morfologia, Instituto de Ciências Biológicas, Universidade de Brasília, Brasília, DF, Brazil.

[4] Departamento de Farmácia, Faculdade de Ciências da Saúde, Universidade de Brasília, Brasília, DF, Brazil.

[5] Laboratório de Microscopia e Microanálises, Departamento de Biologia Celular, Instituto de Ciências Biológicas, Universidade de Brasília, Brasília, DF, Brazil.

[6] Instituto de Física, Universidade de São Paulo, São Paulo, SP, Brazil.

[7] Instituto de Física, Universidade de Brasília, Brasília, DF, Brazil.

[8] Núcleo de Pesquisa em Doenças Negligenciadas, Universidade Brasil, São Paulo, SP, Brazil.

[†] These authors contributed equally to this work.

***** Author for correspondence: jplongo82@gmail.com (J.P.F.L.); moraesnpdn@gmail.com (J.d.M.)

Supporting Information

Table S1. Composition of the mixtures used to define a better SNEDDS formulation.

	**Kolliphor HS 15**	**SPAN 80**	**Castor Oil**	**Praziquantel**
**SNEDDS 1**	-	72.7%	27.3%	70 mg/g
**SNEDDS 2**	18.2%	54.5%	27.3%	70 mg/g
**SNEDDS 3**	36.3%	36.4%	27.3%	70mg/g
**SNEDDS 4**	54.5%	18.2%	27.3%	70 mg/g
**SNEDDS 5**	72.7%	-	27.3%	70 mg/g

Values presented correspond to the average of 15 readings repeated 3 times.

Table S2. Stability of Potential Zeta SNEDDS BLANCK

	**pH 6.8**				**pH 1.2**			
**ZETA (mV)**	**Time 0**	**1 hour**	**2 hours**	**24 hours**	**Time 0**	**1 hour**	**2 hours**	**24 hours**
1/1	-	-	-	-	-	-	-	-
1/10	-33.7	-34.7	-36.3	-38.9	-2.6	-1.4	-0.6	-2.9
1/20	-40.1	-36.5	-37.	-39.6	-3.4	-0.07	-0.7	-0.1
1/30	-39.9	-37.9	-45.3	-46.2	-3.7	-2.7	-2.9	-0.2
1/50	-50.8	-42.4	-43.2	-48.8	0.19	-0.20	-0.8	-0.6
1/100	-50.3	-45.6	-44.8	-50.1	-2.3	-0.9	-0.1	-14.0

Values presented correspond to the average of 15 readings repeated 3 times.

Table S3. Stability of Potential Zeta SNEDDS PZQ

	**pH 6.8**				**pH 1.2**			
**ZETA (mV)**	**Time 0**	**1 hour**	**2 hours**	**24 hours**	**Time 0**	**1 hour**	**2 hours**	**24 hours**
1/1	-	-	-	-	-	-	-	-
1/10	-27.6	-27.3	-27.8	-29.3	0.09	-0.07	-0.11	-0.6
1/20	-30.5	-30.2	-27.9	-27.8	-2.4	-0.07	-2.0	-2.2
1/30	-33.5	-33.5	-39.2	-41.1	0.26	-6.4	-0.16	-4.3
1/50	-37.3	-32.7	-34.9	-42.5	-8.7	-0.002	-10.3	-11.8
1/100	-34.7	-41.5	-43.5	-40.1	-0.07	-22.3	0.2	-20.7

Values presented correspond to the average of 15 readings repeated 3 times.

Table S4. DSC results for PZQ melting peak (°C) and drugs crystallinity (%) of PZQ as supplied and its physical mixtures. Together with TGA data for mass loss (%) occurring up to 500.

Sample	DSC			TGA	
	**Melting peak (*°C*)**	**Enthalpy (*J/g*)**	**Crystallinity (%)**	***Decomposition range (°C)***	***Mass loss (%)***
PZQ	*139.8*	*-124.5*	*100*	*210 –347*	*97.4*
PF1	113.6	-29.3	47.1	222 – 378	91.5
PF2	123.5	-29.1	46.8	216 – 500	97.1
PF3	112.4	-29.2	46.8	207 – 438	90.0
PF4	116.7	-31.3	50.3	230 – 500	94.9

Values presented correspond to the average of 15 readings repeated 3 times.

Table S5. Stability of SNEDDS BLANCK and SNEDDS PZQ in SFG

SNEDDS BLANCK in Pepsin				***SNEDDS PZQ in Pepsin***			
Time (Min.)	*Size (nm)*	*PDI*	*Zeta Potential (mV)*	***Time (Min.)***	*Size (nm)*	*PDI*	*Zeta Potential (mV)*
**0**	*190.3*	*0.2*	*-2.64*	**0**	*169.1*	*0.2*	*0.30*
**5**	*201.5*	*0.2*	*-1.41*	**5**	*201.0*	*0.2*	*-2.99*
**10**	*199.2*	*0.2*	*-2.86*	**10**	*199.0*	*0.2*	*-2.94*
**15**	*205.8*	*0.2*	*-2.90*	**15**	*204.0*	*0.2*	*-2.71*
**30**	*205.0*	*0.2*	*-1.43*	**30**	*199.0*	*0.2*	*-2.21*
**60**	*203.0*	*0.1*	*-0.88*	**60**	*214.0*	*0.2*	*-0.14*
**90**	*210.0*	*0.2*	*-0.75*	**90**	*210.0*	*0.2*	*-0.04*
**120**	*213.2*	*0.3*	*-0.70*	**120**	*212.0*	*0.2*	*0.03*

Values presented correspond to the average of 15 readings repeated 3 times.

Table S6. Stability of SNEDDS BLANCK and SNEDDS PZQ in SFI

SNEDDS BLANCK in Pancreatin				***SNEDDS PZQ in Pancreatin***			
Time (Min.)	*Size (nm)*	*PDI*	*Zeta Potential (mV)*	***Time (Min.)***	*Size (nm)*	*PDI*	*Zeta Potential (mV)*
**0**	*200.0*	*0.2*	*-23.14*	**0**	*196.5*	*0.2*	*-27.27*
**5**	*214.0*	*0.2*	*-25.30*	**5**	*215.9*	*0.3*	*-25.81*
**10**	*201.0*	*0.4*	*-25.50*	**10**	*227.4*	*0.3*	*-26.57*
**15**	*224.0*	*0.4*	*-24.40*	**15**	*224.9*	*0.3*	*-25.98*
**30**	*226.0*	*0.2*	*-25.07*	**30**	*217.3*	*0.3*	*-27.11*
**60**	*237.5*	*0.3*	*-46.29*	**60**	*256.0*	*0.2*	*-45.11*
**90**	*260.8*	*0.3*	*-44.82*	**90**	*272.6*	*0.2*	*-47.72*
**120**	*277.4*	*0.3*	*-43.42*	**120**	*306.6*	*0.2*	*-42.84*

Values presented correspond to the average of 15 readings repeated 3 times.

Table S7. Release kinetic equations of SNEDDS PZQ at pH 1.2.

Kinect Model	Equation	R^2	AIC	K_1	K_2	m	n
**Zero-Order**	$Q = Q_0 - k_0 t$	1	0.009	-	0	-	-
**First-Order**	$\ln Q_t = -kt + \ln Q_0$	0.99	0.007	0		-	-
**Peppas-Sahlin**	$M_t/M_\infty = k_1 t^m + k_2 t^{2m}$	0.98	0.0016	0	0.022	0	-
**Korsmeyer-Peppas**	$M_t/M_\infty = kt^n$	0.97	0.059	-	-	-	0.02
**Higuchi**	$M_t/M_\infty = kt^{0.5}$	0.94	0.068	-	-	-	-
**Hixson-Crowell**	$(1 - M^t/M^\infty)^{1/3} = 1 - kt$	1	0.013	-	-	-	-

Table S8. Release kinetic equations of SNEDDS PZQ at pH 6.8.

Kinect Model	Equation	R^2	AIC	K_1	K_2	m	n
**Zero-Order**	$Q = Q_0 - k_0 t$	1	0.004	-	0.007	-	-
**First-Order**	$\ln Q_t = -kt + \ln Q_0$	1	0.006	0.008		-	-
**Peppas-Sahlin**	$M_t/M_\infty = k_1 t^m + k_2 t^{2m}$	0.99	0	0	0.006	0	-
**Korsmeyer-Peppas**	$M_t/M_\infty = kt^n$	0.98	0.009	-	-	-	0.010
**Higuchi**	$M_t/M_\infty = kt^{0.5}$	0.94	0.056	-	-	-	-
**Hixson-Crowell**	$(1 - M^t/M^\infty)^{1/3} = 1 - kt$	1	0.005	-	-	-	-

Video S1: Dispersibility of the SNEDDS (22 grams) formulation in 200 ml of water.

Rheology

Result

Rheological analysis revealed that both the oily phase with and without the drug exhibited characteristics of a Newtonian fluid, demonstrating consistent viscosity regardless of shear rate. However, upon formation of the nanoparticle, a non-Newtonian behavior was observed, specifically resembling a shear thickening fluid, irrespective of drug presence (Figure S1). Hence, while the viscosity of the oil phase remained constant, that of the nanoparticle formulation increased proportionally with shear.

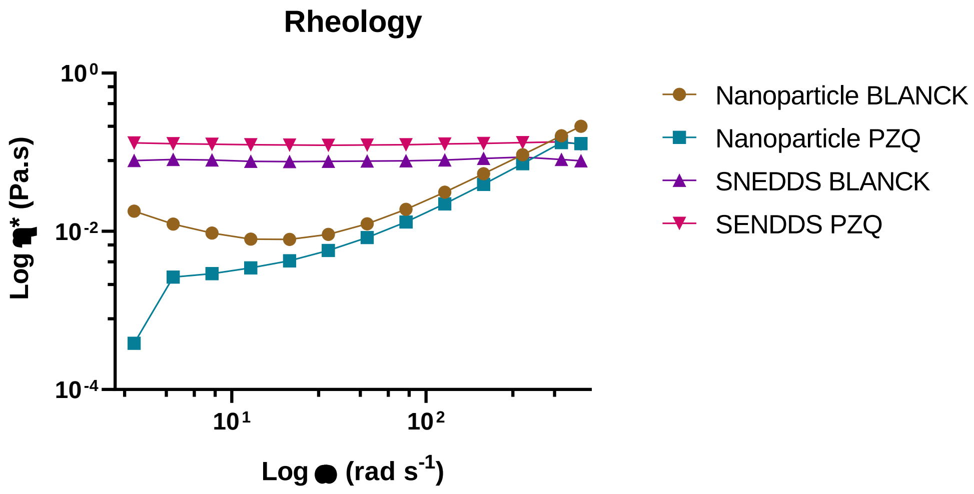

Figure S1. Rheological Properties of Nanoparticle and SNEDDS Formulations. Complex viscosity (η*) as a function of frequency (ω) at 25°C, measured via controlled-stress rheometry. Nanoparticle BLANCK (brown circles) and PZQ (blue squares) formulations exhibit shear-thinning behavior, with viscosity decreasing as frequency increases, suggesting nanoparticle rearrangement under shear. In contrast, SNEDDS BLANCK (purple upward-pointing triangles) and SENDDS PZQ (pink downward-pointing triangles) demonstrate Newtonian-like behavior with relatively constant viscosity across the frequency range. Data represents the average of three independent measurements (n=3), with error bars indicating standard deviation. These flow properties offer key insights into the formulations' behavior under stress, essential for predicting their stability and performance in drug delivery.

Discussion

In rheological assays, the transition from a Newtonian to a non-Newtonian behavior in the nanoparticle system is a critical observation. Newtonian viscosity is characterized by a fluid's ability to retain a consistent viscosity, irrespective of the changes in shear rate applied to it. This means that the oil phase behaves according to Newton's law of viscosity, where the shear stress is proportional to the shear rate. However, the dynamic processes involved in nanoparticle formation and drug encapsulation significantly alter this scenario. During these processes, the stress forces applied bring the particles closer together so that they begin to hinder each other's movement. This interaction effectively increases the system's viscosity[1]. As a result, the intra-molecular interactions are converted to inter-molecular interactions upon the imposition of shear rates[2]. This phenomenon can play a pivotal role in enhancing the efficacy of drug delivery systems. A fluid that becomes more viscous while preserving its nanometric structure can offer

extended retention in the gastrointestinal tract, thereby providing a more controlled and gradual release of the drug.

Supporting Information

Praziquantel pharmacokinetics

Drug determination in plasma

PZQ was quantified following a bioanalytical method using reversed-phase high-performance liquid chromatography (HPLC) with ultraviolet detection. Samples were analyzed by Shimadzu HPLC (Kyoto, Japan), model LC 20-AD, equipped with diode array detector (SPD-20A), pump (LC-20D), degasser (DGU-20A3), automatic injector (model 9SIL-20AD), and oven (model CTO-20AS). Linearity ($y = 158662x + 816{,}34$; $r^2 = 0.999$) was determined in the range of 0,5 – 20 mg L-1, where y is the peak area and x is the drug concentration in µg mL− 1. The wavelength used for detection was 210 nm. The stationary phase was a Shimadzu C18 reversed-phase column (150 × 4.5 mm × 5 µm). The mobile phases consisted of 55% water and 45% acetonitrile operating at a flow of 0.7 mL min-1. The method showed an adequate retention time for identifying the drug (9.55 min) with a limit of detection (LOD) and the limit of quantification (LOQ) of 0.07 and 0.21 mg L-1, respectively. The analysis of raw data and peak integrations were performed using the software LabSolutions (Shimadzu, Kyoto, Japan).

Quantification of drug in plasma

Mice subcutaneous infected with ~30 cercarie were treated with PZQ and SNEDDS-PZQ 400 mg/kg in different times: 15, 30, 60, 120, 240, 360, 480 and 1440 minutes. Animals were euthanized and the blood collected by mesenteric vein. Blood samples were centrifugated and plasma samples were kept with lithium heparin in -80 °C until quantified. After storage, the animals' plasma was subjected to an ultrasound bath for 25 minutes, with heating. at 37º C. Next, the PZQ in the collected plasma was quantified as described previously.

Results

The PZQ plasm quantification was plotted in a curve graph and it is represented in Figure S2, as following. The study compares the pharmacokinetic profiles of two formulations: SNEDDS-PZQ and standard PZQ. Figure S2 details the plasma concentration of each formulation over a 1440-minute timeframe. The SNEDDS-PZQ formulation exhibited a higher initial plasma concentration with a rapid decrease within the first 200 minutes, followed by a stabilized phase. In contrast, the conventional PZQ formulation showed a consistent, gradual decline in plasma concentration throughout the study period.

These findings indicate that the SNEDDS-PZQ configuration may enhance drug delivery efficiency and prolong plasma concentration, potentially improving therapeutic outcomes. The

improved bioavailability of SNEDDS-PZQ underscores its potential advantages over the traditional PZQ formulation, suggesting further investigation into its clinical applications.

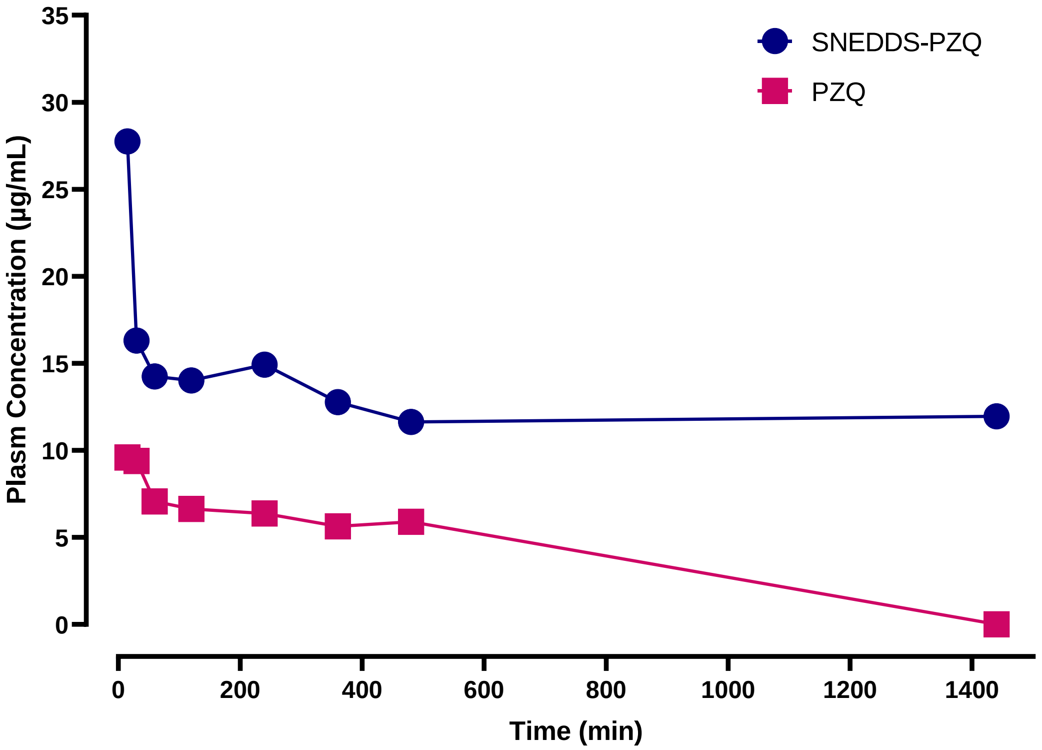

Figure S2: This figure illustrates the plasma concentration of two formulations, SNEDDS-PZQ (blue circles) and PZQ (pink squares), measured in micrograms per milliliter (µg/mL) over a period of 1440 minutes.

References

1. Minghai W., Kun L., Li S. Shear thickening fluids and their applications. *Materials & Design*. 2022; **216**: 110570.
2. Jaishankar A., Wee M., Matia-Merino L., et al. Probing hydrogen bond interactions in a shear thickening polysaccharide using nonlinear shear and extensional rheology. *Carbohydr. Polym*. 2015; **123**: 136-145.
